# Alleviation effect of arbutin on oxidative stress generated through tyrosinase reaction with l-tyrosine and l-DOPA

**DOI:** 10.1186/1471-2091-15-23

**Published:** 2014-10-09

**Authors:** Mika Tada, Masahiro Kohno, Yoshimi Niwano

**Affiliations:** 1 Center for General Education, Tohoku Institute of Technology, Sendai, Japan; 2 Graduate School of Bioscience and Biotechnology, Tokyo Institute of Technology, Yokohama, Japan; 3 Tohoku University Graduate School of Dentistry, 4-1 Seiryo-machi, Aoba-ku, Sendai 980-8575, Japan

**Keywords:** Hydroxyl radical, Tyrosinase reaction, Arbutin

## Abstract

**Background:**

Hydroxyl radical that has the highest reactivity among reactive oxygen species (ROS) is generated through l-tyrosine-tyrosinase reaction. Thus, the melanogenesis might induce oxidative stress in the skin. Arbutin (*p*-hydroxyphenyl-β-d-glucopyranoside), a well-known tyrosinase inhibitor has been widely used for the purpose of skin whitening. The aim of the present study was to examine if arbutin could suppress the hydroxyl radical generation via tyrosinase reaction with its substrates, l-tyrosine and l-DOPA.

**Results:**

The hydroxyl radical, which was determined by an electron spin resonance-spin trapping technique, was generated by the addition of not only l-tyrosine but l-DOPA to tyrosinase in a concentration dependent manner. Arbutin could inhibit the hydroxyl radical generation in the both reactions.

**Conclusion:**

It is presumed that arbutin could alleviate oxidative stress derived from the melanogenic pathway in the skin in addition to its function as a whitening agent in cosmetics.

## Background

Native human melanin consists of eumelanin and pheomelanin, and eumelanin is found in almost every type of human skin [1,2]. In the skin, melanin synthesized in melanocytes, which are located in the basal layer and hair bulbs, transfers to keratinocytes. Melanin in keratinocytes acts as a photoprotector through body coloration and scavenging reactive oxygen species such as superoxide anion and singlet oxygen [3-8]. Despite the photoprotective role of melanin, many cosmetics have been developed to prevent melanin formation in the skin because of aesthetic satisfaction by whitening ability. Of these, inhibitor of tyrosinase, which is a pivotal enzyme for melanin synthesis [9], has been used as a major ingredient of cosmetics [10-14]. Tyrosinase, an enzyme which contains dinuclear copper ions at the active site [15-17], catalyzes two distinct reactions of melanin synthesis [18], the hydroxylation of a monophenol and the conversion of an *o*-diphenol to the corresponding *o*-quinone, indicating that l-tyrosine is hydroxylated to l-DOPA, which is in turn converted to dopaquinone. Our previous study applying electron spin resonance (ESR)-spin trapping method revealed that hydroxyl radicals are generated through L-tyrosine-tyrosinase reaction [19], so that we assumed that dicopper-peroxide intermediates formed during the catalytic process of l-tyrosine to dopaquinone possibly decay to produce the hydroxyl radical through an internal electron transfer from the ligand. This suggests that tyrosinase inhibitors might contribute to alleviate the oxidative damage of the skin by inhibiting hydroxyl radical generation via the enzyme reaction.

Arbutin (*p*-hydroxyphenyl-β-d-glucopyranoside), a well-known tyrosinase inhibitor, which can be extracted from plants, has been widely used for the purpose of skin whitening [20]. Regarding the molecular base mechanisms of arbutin, it was reported that arbutin inhibits not only the oxidation of l-DOPA but the hydroxylation of l-tyrosine [21,22]. With regard to skin-whitening effect of arbutin in relation to melanogenesis, it was reported that arbutin showed no effect on the differentiation of melanocytes while hydroquinone used as a skin-whitening agent downregulated the differentiation [23]. Besides tyrosinase inhibition, it was reported that arbutin has anti-inflammatory effect [24], it is expected that arbutin could alleviate inflammation in the skin exposed to ultraviolet (UV) light.

The purpose of the present study was to examine if a tyrosinase inhibitor could suppress the hydroxyl radical generation via tyrosinase reaction with its substrates, l-tyrosine and l-DOPA. In the study, arbutin was used as a representative tyrosinase inhibitor.

## Results and discussion

**Figure 1:**
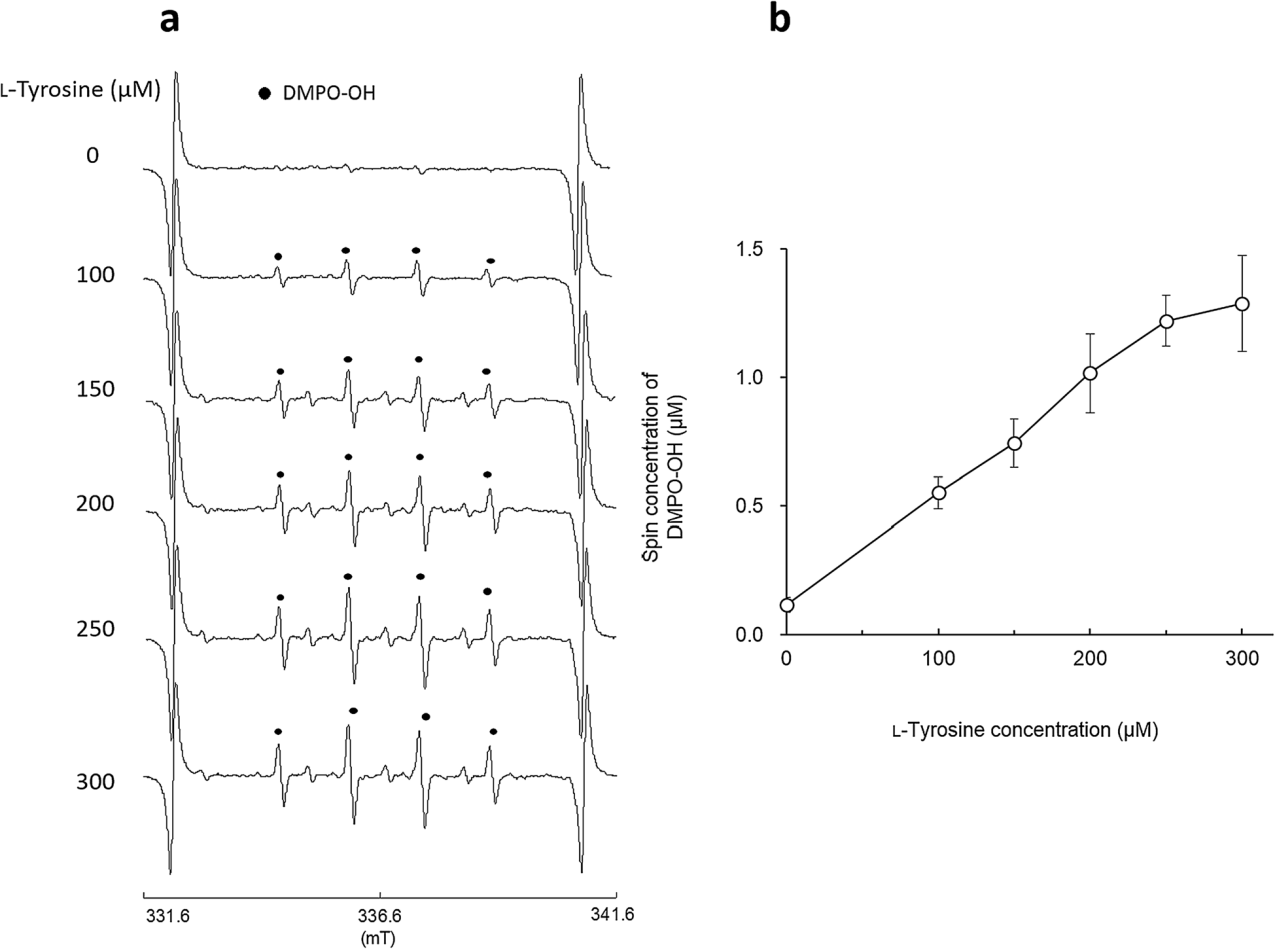
**Hydroxyl radical formation by L-tyrosine-tyrosinase reaction. (a)** Representative ESR spectra with different concentrations of l-tyrosine, and **(b)** the yield of DMPO-OH (mean ± SD, n=3 or 4).

**Figure 2:**
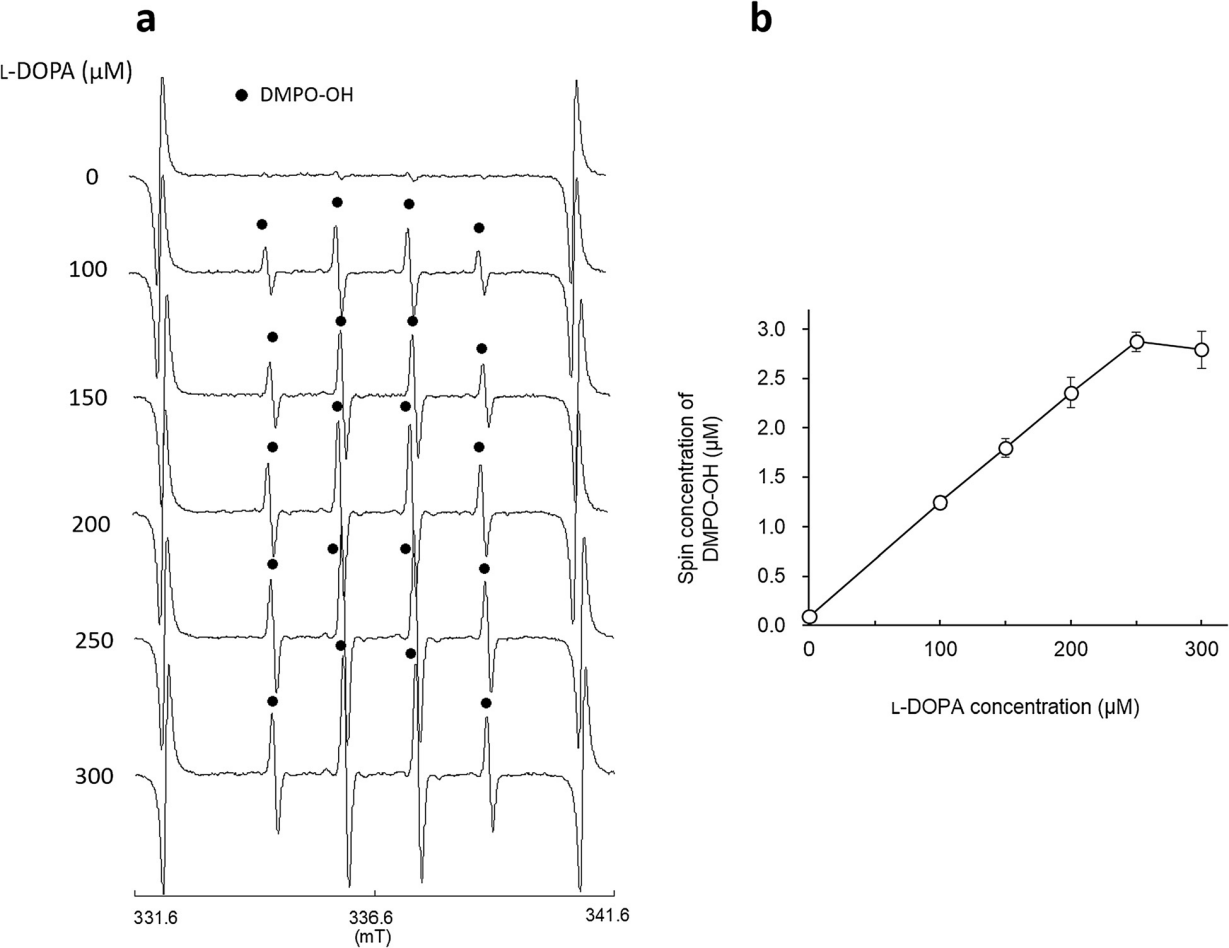
**Hydroxyl radical formation by L-DOPA-tyrosinase reaction. (a)** Representative with different concentrations of l-DOPA, and **(b)** the yield of DMPO-OH (mean ± SD, n=3).

**Figure 3:**
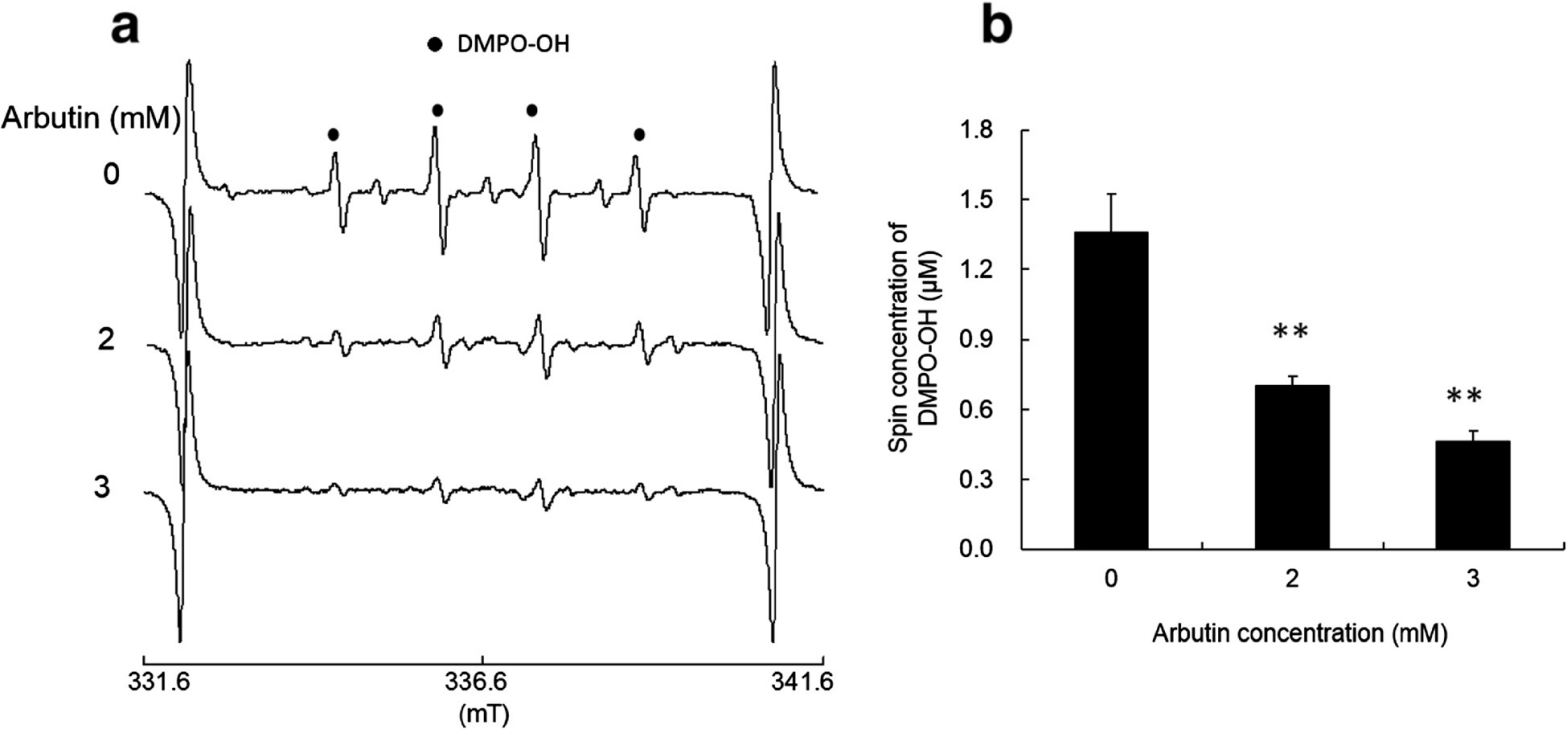
**Effect of arbutin on hydroxyl radical formation. (a)** Representative ESR spectra obtained by the addition of arbutin to the l-tyrosine-tyrosinase solution, and **(b)** the yield of DMPO-OH (mean + SD, n=3). Significant differences from the control are shown as P<0.01 (**).

**Figure 4:**
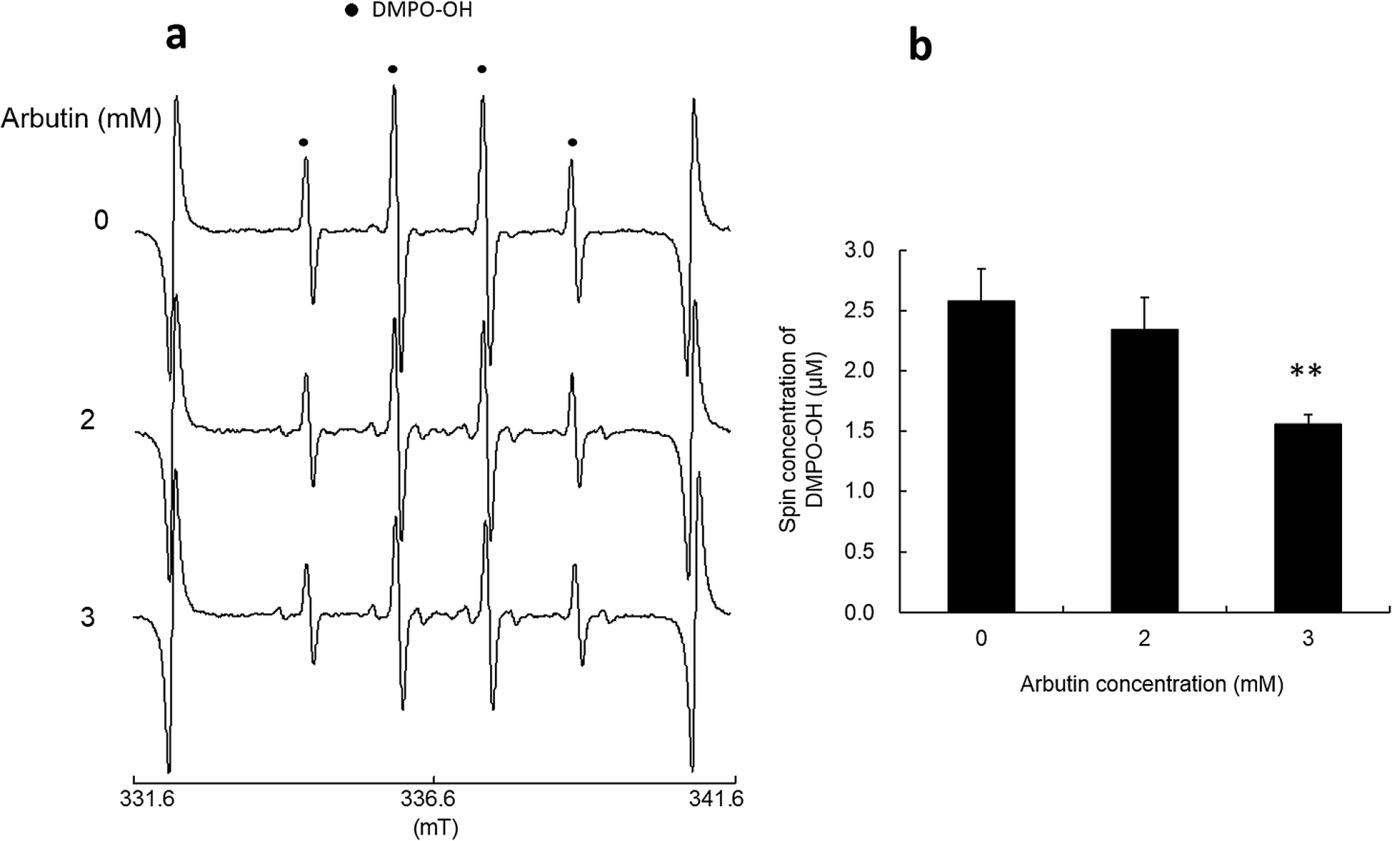
**Effect of arbutin on hydroxyl radical formation. (a)** Representative ESR spectra obtained by the addition of arbutin to the l-DOPA-tyrosinase solution, and **(b)** the yield of DMPO-OH (mean + SD, n=2 or 3). Significant difference from the control is shown as P<0.01 (**).

As reported in our previous study, clear ESR signals were obtained by the l-tyrosine-tyrosinase reaction. Representative ESR spectra obtained from l-tyrosine-tyrosinase reaction with different concentrations of l-tyrosine are summarized in Figure 1a. Typical ESR spectrum (intensity ratio, 1:2:2:1) was assigned to 5,5-dimethyl-1-pyrroline *N*-oxide (DMPO)-OH, a spin adduct derived from hydroxyl radical (hyperfine coupling constant, aN =1.49; aH =1.49 mT) as reported in a previous study [19], and signal intensity of DMPO increased with the concentrations of l-tyrosine, which is also supported by the calculated yield of DMPO-OH as shown in Figure 1b. When l-DOPA was added as a substrate to the reaction mixture containing tyrosinase, similar ESR spectra to those in Figure 1a were obtained (Figure 2a), and the resultant DMPO-OH yield also increased with the concentration of l-DOPA. Figure 3a and b show the representative ESR spectra obtained by the addition of arbutin to the l-tyrosine-tyrosinase solution and the yield of DMPO-OH, respectively. Arbutin clearly lowered the signal intensity of DMPO-OH, suggesting that generation of hydroxyl radicals via l-tyrosine-tyrosinase reaction was inhibited by arbutin in a concentration dependent manner, and IC50 (the concentration which showed 50% inhibition) calculated by using an approximate formula was 1.7 mM. Figure 4a and b show the representative ESR spectra obtained by the addition of arbutin to the l-DOPA-tyrosinase solution and the yield of DMPO-OH, respectively. When L-DOPA was used as a substrate, the signal intensity of DMPO-OH was also reduced (IC50 was 2.6 mM), suggesting that arbutin has an ability to reduce the generation of hydroxyl radicals through tyrosinase reaction even when l-DOPA was used as a substrate for the enzyme.

**Figure 5:**
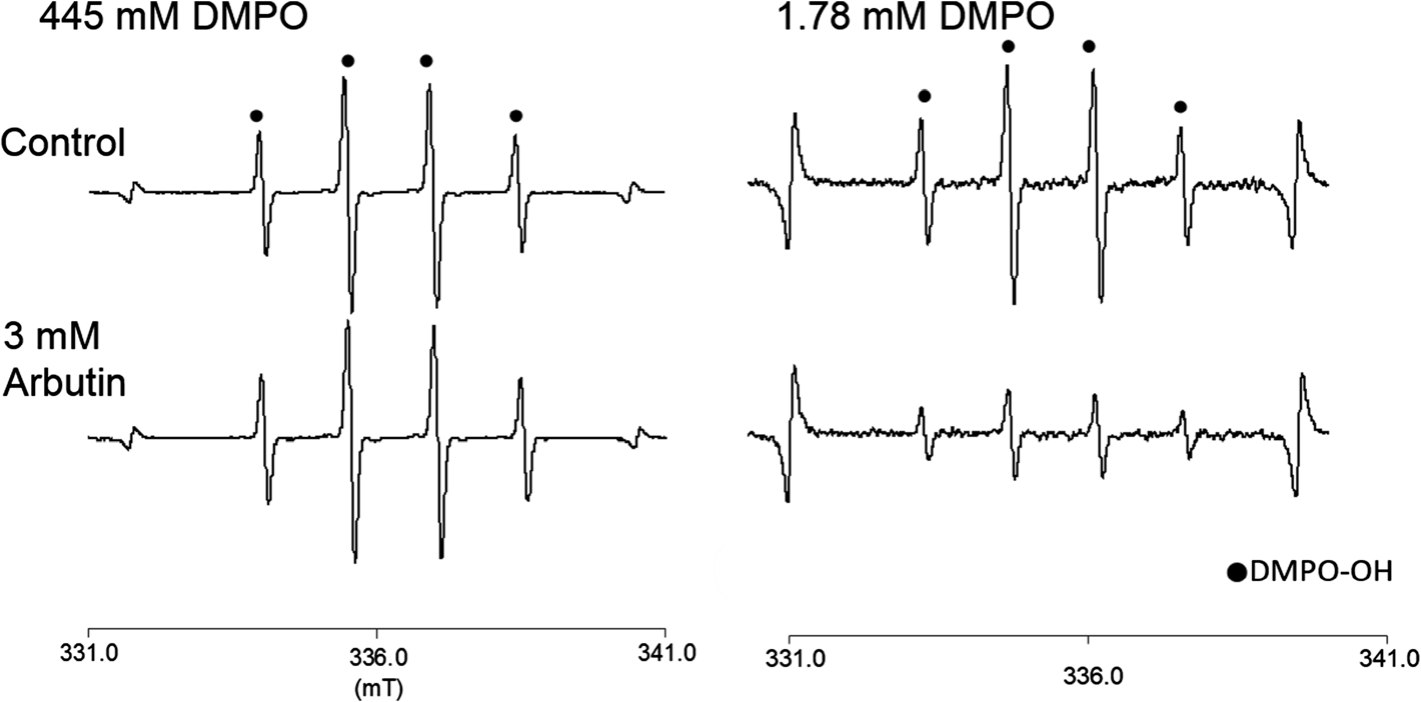
Representative ESR spectra obtained by the addition of arbutin to a Fenton reaction.

Since it was reported that arbutin inhibits not only the oxidation of l-DOPA but the hydroxylation of l-tyrosine [21,22], we hypothesized that arbutin has an ability to suppress the hydroxyl radical formation through the process of tyrosinase reaction to produce melanin. The results of the present study clearly support the hypothesis. Since it was reported that arbutin exhibited antioxidant activity as determined by the 1,1-diphenyl-2-picrylhydrazyl (DPPH) radical scavenging assay but that it was not strong compared to that of antioxidant vitamins, hydroquinone, and caffeic acid [25,26]. Thus, we examined the effect of arbutin on the hydroxyl radical generated by a Fenton reaction. Figure 5 shows the representative ESR spectra obtained by the Fenton reaction with 1.78 mM DMPO or 445 mM DMPO. As a result, when 445 mM DMPO that was the same concentration used in the ESR-spin trapping assay described above was used as a spin trapping agent, no reduction in the signal intensity of DMPO-OH was found. Whilst the reduction in the signal intensity of DMPO-OH was found when the concentration of DMPO was lowered to 1.78 mM. Thus, although arbutin reduced DMPO-OH level, the effect was very weak, suggesting that a goodly portion of the reduced amount of hydroxyl radicals observed in the present study was likely attributable to the inhibition of tyrosinase reaction. Regarding the possible involvement of other ROS, since no relevant ESR signal to superoxide anion (DMPO-OOH) was observed in the ESR spectra obtained in the present study and our previous study also revealed that neither superoxide dismutase nor catalase affected the amount of DMPO-OH [19], it is suggested that neither superoxide anion nor hydrogen peroxide is involved in the hydroxyl radical generation by the tyrosinase reaction. As for singlet oxygen, it was reported that singlet oxygen is the main reactive species formed by UVA irradiation on the skin, we will further examine the involvement of singlet oxygen by using an appropriate model with UVA irradiation [27,28]. From these, it is presumed that arbutin could alleviate oxidative stress derived from the melanogenic pathway in the skin in addition to its function as a whitening agent in cosmetics. The enzymatic oxidation of l-tyrosine to melanin is of considerable importance because melanin has many functions such as light absorption and scattering in terms of skin protection from photo-aging. On the other side of the coin, the enzyme reaction could cause oxidative damage in the skin via the hydroxyl radical which has the highest reactivity among ROS [29]. Regarding UV-mediated oxidative stress, in addition to singlet oxygen generated upon exposure to UVA [27,28] as described above, it was reported that specific ROS, including superoxide anion, hydrogen peroxide, and hydroxyl radical are enhanced in keratinocytes treated with UVB, and superoxide anion and hydroxyl radical are the main ROS contributing to oxidative stress in the early phase after UVB treatment [30]. Besides ROS formation, UV irradiation also induces melanogenesis. For instance, cocultures of keratinocytes and melanocytes show the importance of cell communication in the melanogenic response through endogenous mediator, nitric oxide upon irradiation by UV [31]. Thus, it seems to be of importance to consider the balance between the UV- and the melanogenesis pathway-mediated oxidative stress because melanogenesis is induced to alleviate the effect of UV. In terms of alleviating oxidative stress in the skin, therefore, combination of a tyrosinase inhibitor such as arbutin and a UV-absorbing agent would be very effective. In the present study, the effect of arbutin was examined in in vitro enzyme reaction system. To validate the alleviation of oxidative stress by arbutin, in vitro melanocyte study as well as in vivo study should be conducted. It was reported that arbutin inhibited the tyrosinase activity of cultured human melanocytes at noncytotoxic concentrations [20], so that we expect that arbutin could alleviate the oxidative stress induced by tyrosinase reaction at cellular level. However, to detect the effect of arbutin, it would be better for the cells to be under the condition of accelerated melanogenesis which could be induced by UV irradiation as reported previously [32]. We will further examine the effect of arbutin on the oxidative stress caused by accelerated melanogenesis in in vitro melanocytes as the next step.

## Conclusion

The hydroxyl radical generation via tyrosinase reaction with either L-tyrosine or L-DOPA was reduced by a tyrosinase inhibitor, arbutin. Thus, it is expected that tyrosinase inhibitors such as arbutin could alleviate oxidative stress derived from the melanogenic pathway in the skin in addition to its function as a whitening agent in cosmetics.

## Methods

### Test materials and reagents

Reagents were purchased from the following sources: l-Tyrosine, l-DOPA and phosphate buffer solution (PB, pH 6.5) from Wako Pure Chemicals (Osaka, Japan); tyrosinase (from mushroom) from Sigma-Aldrich Corp. (St. Louis, MO); DMPO from Labotec (Tokyo, Japan); arbutin from LKT Laboratories, Inc. (St. Paul, MN). All other reagents used were of analytical grade.

### ESR-spin trapping determinations of hydroxyl radicals generated by tyrosinase reaction with l-tyrosine and l-DOPA

Tyrosinase was dissolved in PB to be 100 U/ml. l-Tyrosine was dissolved in PB to be 200 mM. Then 1 mM l-tyrosine solution was prepared by mixing 5 μl of 200 mM l-tyrosine solution with 5 μl of 1 M NaOH and 990 μl of 0.2 M PB. l-DOPA was also dissolved in 1 M HCl to be 200 mM. Then 1 mM l-DOPA solution was prepared by mixing 5 μl of 200 mM l-DOPA solution with 5 μl of 1 M NaOH and 990 μl of 0.2 M PB. Formulated concentrate of DMPO (8.9 M) was used. The reaction mixture was prepared to contain different volume of substrate (1 mM l-tyrosine or 1 mM l-DOPA), 10 μl of 8.9 M DMPO, 4 μl of 100 U/μl tyrosinase and 0.2 M PB which was added to adjust a total volume of 200 μl. Immediately after mixing the mixture was transferred to an ESR spectrometry cell, and the ESR measurement was started after 45 s. The measurement conditions of ESR (JES-FA-100, JEOL, Tokyo, Japan) were as follows: field sweep, 330.80-340.80 mT; field modulation frequency, 100 kHz; filed modulation width, 0.07 mT; amplitude, 400; sweep time, 1 min; time constant, 0.1 s; microwave frequency, 9.430 GHz; microwave power, 5 mW. In the study where the effect of arbutin on the hydroxyl radical generation was examined, 100 mM arbutin dissolved in ultrapure water was diluted 10 times with 0.2 M PB. The reaction mixture was prepared to contain different volume of 10 mM arbutin, 60 μl of substrate (1 mM l-tyrosine or 1 mM l-DOPA), 10 μl of 8.9 M DMPO, 4 μl of 100 U/μl tyrosinase and 0.2 M PB which was added to adjust a total volume of 200 μl. The concentrations of arbutin used in the study were decided by the enzyme assay for tyrosinase where dopachrome formation was monitored at 475 nm. Arbutin at a concentration of 1.5 mM or more clearly inhibited dopachrome formation (data not shown). To further examine if arbutin has an ability to scavenge directly hydroxyl radicals, effect of arbutin on the hydroxyl radical generated by a Fenton reaction. The reaction mixture was prepared to contain 3 mM arbutin, 0.5 mM H_2_O_2_, 0.05 mM FeSO_4_, and 1.78 mM or 445 mM DMPO, and was subjected to ESR analysis.

### Statistical analysis

In the experiments where the effect of arbutin was examined, statistical significances (p < 0.05) in the yield of DMPO-OH were assessed by Dunnett’s multiple comparison test.

## Competing interests

Authors declare that no competing interests.

## Authors’ contributions

MT, MK, and YN conceived the study, carried the sample preparation, and ESR determinations. YN drafted the manuscript. All authors read and approved the final manuscript.
